# EIF3J-AS1 promotes glioma cell growth via up-regulating ANXA11 through sponging miR-1343-3p

**DOI:** 10.1186/s12935-020-01487-2

**Published:** 2020-09-03

**Authors:** Jianguo Qi, Zhengrui Wang, Zhensheng Zhao, Lijun Liu

**Affiliations:** 1 Department of Neurosuigery, The Third People’s Hospital of Jinan, Jinan, 250101 Shandong China; 2 Department of Neurosurgery, Chengyang People’s Hospital of Qingdao, Qingdao, 266109 Shandong China; 3 Department of Hyperbaric Oxygen Therapy, Yidu Central Hospital of Weifang, Weifang, 262500 Shandong China; 4 Department of Neurosurgery, Xiangyang No.1 People’s Hospital Affiliated to Hubei University of Medicine, NO.15 Jiefang Road, Fancheng District, Xiangyang, 441000 Hubei China

**Keywords:** Glioma, EIF3J-AS1, miR-1343-3p, ANXA11

## Abstract

**Background:**

Glioma is one prevalent malignant tumor originates from the central nervous system. Dysregulation of long non-coding RNAs (lncRNAs) has been found to be a molecular signature behind the pathology of a variety of cancers, including glioma. EIF3J antisense RNA 1 (EIF3J-AS1) is a novel lncRNA, whose performance in carcinogenesis has been unfolded. Nevertheless, the role of EIF3J-AS1 has never been investigated in glioma.

**Methods:**

qRT-PCR analysis was adopted to evaluate the relative levels of RNAs. In vitro functional assays, including colony formation, EdU, TUNEL and caspase-3/8/9 activity assays were conducted to study the impacts of EIF3J-AS1 on glioma. Dual-luciferase activity assays, RNA pull down assay and RIP assay were performed to elucidate molecular interplay among genes.

**Results:**

EIF3J-AS1 was overexpressed in glioma cell lines. Knockdown of EIF3J-AS1 hampered glioma malignant phenotypes. MiR-1343-3p could bind to EIF3J-AS1. Moreover, miR-1343-3p targeted Annexin A11 (ANXA11) in its 3′UTR region. Mechanistically, EIF3J-AS1 relieved ANXA11 from miR-1343-3p silencing in the EIF3J-AS1/miR-1343-3p/ANXA11 RNA induced silencing complex (RISC), thus eliciting promoting effects on glioma progression. MiR-1343-3p inhibitor and ANXA11 overexpression offset the inhibitory impacts of EIF3J-AS1 silencing on glioma development.

**Conclusion:**

EIF3J-AS1/miR-1343-3p/ANXA11 axis significantly affected biological behaviors in glioma, suggesting new therapeutic target for glioma treatment.

## Background

Glioma remains the most frequently diagnosed primary tumor and lethal form of brain tumors [1]. The most common histologic subtypes of glioma are astrocytoma and oligodendroglioma [2]. Despite multimodal therapeutic strategy, such as chemotherapy, radiotherapy and other adjuvant therapy have been combined to combat glioma over the last decade, the prognosis of glioma patients remains unoptimistic [3, 4]. Lack of efficient and novel molecular target in clinical treatment is a vital reason for the unsatisfactory outcome [5]. Therefore, exploring molecular mechanism of glioma is of vital significance.

Cancer transcriptome is featured in aberrant expression of both protein-coding and non-coding transcripts. Numerous evidence have indicated that long non-coding RNAs (lncRNAs) played important part in regulating the development of human diseases, even the malignant progression of cancers, including glioma [6–8]. Recently, emerging reports have elaborated that the dysregulated lncRNAs was intimately associated with the pathology of various malignant tumors and may serve as sensitive diagnostic biomarker or treatment target [9, 10].

Notably, growing lncRNAs were found to regulate gene expression level via acting as competitive endogenous RNAs (ceRNA) in a post-transcriptional manner [11, 12]. In the classical model of ceRNA network, lncRNAs that harbor miRNA-response elements (MREs) could sequester miRNAs and offset the repression effects on target messenger RNAs (mRNAs) [13]. This important regulatory mechanism of lncRNAs was gradually gaining increasing attention in anti-cancer research. Similarly, the internal connection among lncRNA, miRNA, and mRNA has been explored in glioma. LncRNA taurine upregulated 1 (TUG1) has been validated to up-regulate vascular endothelial growth factor A (VEGFA) in promoting angiogenesis in human glioblastoma via sponging shared miR-299 [14]. LncRNA EIF3J antisense RNA 1 (EIF3J-AS1) was firstly reported to notably up-regulated in hepatocellular carcinoma (HCC) tumor samples and closely related with recurrence-free survival in HCC [15]. However, its latent biological performance for glioma cells malignant behaviors has never been studied.

Annexin A11 (ANXA11) is a protein-coding gene that encodes a member of the annexin family, a class of calcium-dependent phospholipid-binding proteins. ANXA11 has been found to play a part in the biology of some cancers. For example, it was revealed to regulate gastric cancer proliferation, migration, and invasion via mediating AKT/GSK-3β pathway [16]. However, the molecular relationship between EIF3J-AS1 and ANXA11 remains elusive in glioma.

Collectively, this study was aimed at investigating the role of EIF3J-AS1/miR-1343-3p/ANXA11 axis in glioma growth.

## Methods

### Tissue specimens

Forty glioma patients who diagnosed and underwent primary surgery in the Third People’s Hospital of Jinan between June 2017 and April 2019 enrolled in this study. Normal brain samples were collected from 10 patients who suffered severe head trauma and underwent partial removal of normal brain tissue. None of these participants received any therapies before surgery. This study was conducted based on the Declaration of Helsinki. All patients provided the informed consent and the Ethics Committee of the Third People’s Hospital of Jinan approved current study.

### Cell lines

Five glioma cell lines (A-1235, H4, U251, 42-MG-BA, A-172) and one normal glial cell line (HEB), from the ATCC (Rockville, Maryland), were preserved in the DMEM medium (Invitrogen, Carlsbad, CA) under 5% CO_2_ and 37 °C. 1% antibiotics and 10% FBS were available from Invitrogen for purpose of medium supplements.

### Quantitative reverse transcription polymerase chain reaction (qRT-PCR)

Total RNAs from the cultured cells were acquired with the TRIzol reagent (Invitrogen). Total RNA (1 μg) was employed for reverse transcription as per the user manual (Thermo Fisher, Waltham, MA). qRT-PCR was then performed with the SYBR green Supermix as instructed by manufacturer (Thermo Fisher). The comparative change-in-cycle method (ΔΔCt) was used to calculate the changes in gene expression. GAPDH or U6 served as the standardized gene.

### Transfection

The synthesized shRNAs and NC-shRNAs (Genepharma, Shanghai, China) were obtained to silence EIF3J-AS1 and ANXA11 in A-1235 and U251 cells employing the Invitrogen Lipofectamine2000. In addition, the pcDNA3.1/EIF3J-AS1, pcDNA3.1/ANXA11 and NC-pcDNA3.1, as well as miR-1343-3p mimics/inhibitor and NC mimics/inhibitor, were all procured from Genepharma for purpose of 49 h of plasmid transfection.

### Colony formation assay

Cells of A-1235 and U251 at log phase of growth were reaped and plated into 6-well plates with 800 cells per well. Cells were cultivated for 14-day. After fixing in 4% paraformaldehyde, colonies were counted manually by 0.1% crystal violet staining.

### EdU assay

5 × 10^4^ cells of A-1235 and U251 in 96-well plates were incubated for 2 h in the medium adding EdU, and then fixed for 30 min. According to the direction of EdU assay kit (Ribobio, Guangzhou, China), the proliferative cells were detected. Then nucleus was monitored by DAPI staining and subjected to fluorescent microscope (Leica, Wetzlar, Germany).

### Caspase-3/8/9 activity assay

The activities of caspase-3/8/9 were severally monitored in A-1235 and U251 cells employing Caspase-3/8/9 assay kit (Abcam, Cambridge, MA). After 72 h of incubation under standard condition, cells in 6-well plates were measured by microplate reader at 405 nm.

### TUNEL assay

After transfection, cells of A-1235 and U251 were fixed for 1 h, premeabilized for 2 min on ice, and then washed in PBS. Cells were cultured for 1 h in the dark at 37 °C with 50 μl of TUNEL reaction buffer as per the direction of TUNEL assay kit (Beyotime, Shanghai, China), followed by observing under fluorescent microscope.

### In vivo animal study

Six BALA/C nude mice (4–6 weeks old, 17–20 g), purchased from the Laboratory Animal Center of Jilin University (Changchun, Jilin, China) were grown in constant conditions (temperature: 25–27  °C; humidity: 45–50%). All mice were randomly divided into two groups (three in each group): sh-NC, sh-EIF3J-AS1. Stably transfected cells were detached when the confluence reached to 80–90%. After centrifugation and re-suspension. 1 × 10^7^ cells/mL were injected into the thigh skin of the nude mice. Tumor size were monitored every 4 days. Four weeks later, the nude mice were killed and the tumors were removed for weighing [17].

### Subcellular fractionation

Cells were lysed in cell fractionation buffer, and then centrifuged. After collecting the supernatant, the remaining pellet was rinsed in cell fractionation buffer and centrifuged, then added with cell disruption buffer. The RNAs of cell cytoplasm and cell nucleus were extracted for qRT-PCR analysis.

### Fluorescence in situ hybridization (FISH)

The specific probe of EIF3J-AS1 was available from Ribobio for FISH assay. The fixed cells were treated with protease K, washed in PBS and dehydrated. After denaturation, hybrid solution was added for hybridization purposes overnight, finally Hoechst staining and fluorescence detection were performed.

### RNA pull down

Using Pierce Magnetic RNA–Protein Pull-Down Kit, RNA pull down assay were carried out in A-1235 and U251 cells according to the direction (Thermo Fisher). The cellular protein extracts were used to mix with the biotinylated EIF3J-AS1 or miR-1343-3p probes, and then cultured with magnetic beads at 4 °C for 1 h. Following collecting the pull-downs, qRT-PCR was conducted.

### RNA Immunoprecipitation (RIP) assay

1 × 10^7^ cells of A-1235 and U251 were collected from the RIP lysis buffer, then immunoprecipitated with anti-Ago2 antibody or anti-IgG antibody (Abcam). The precipitates were recovered by beads and subjected to qRT-PCR for relative RNA enrichment.

### Dual-luciferase activity assay

The wild-type and mutant EIF3J-AS1 or ANXA11 fragments covering miR-1343-3p binding sites were synthesized and inserted into the pmirGLO luciferase vector (Promega, Madison, WI). The acquired reporter vectors EIF3J-AS1-WT/Mut and ANXA11-WT/Mut were co-transfected with miR-1343-3p mimics or NC mimics in A-1235 and U251 cells for 48 h. Luciferase assay was achieved by use of Luciferase Reporter Assay System (Promega).

### Statistical analyses

Each assay was implemented in triplicates, and results were shown as mean ± the standard deviation (SD). Student’s *t* test or one-way ANOVA was used for data analysis utilizing the PRISM 6 (GraphPad, San Diego, CA), with p-value below 0.05 as significant level. Pearson correlation test was applied to analyze the expression correlation between RNAs in tissue samples.

## Results

### Upregulation of EIF3J-AS1 in glioma facilitates malignant progression of glioma

**Fig. 1:**
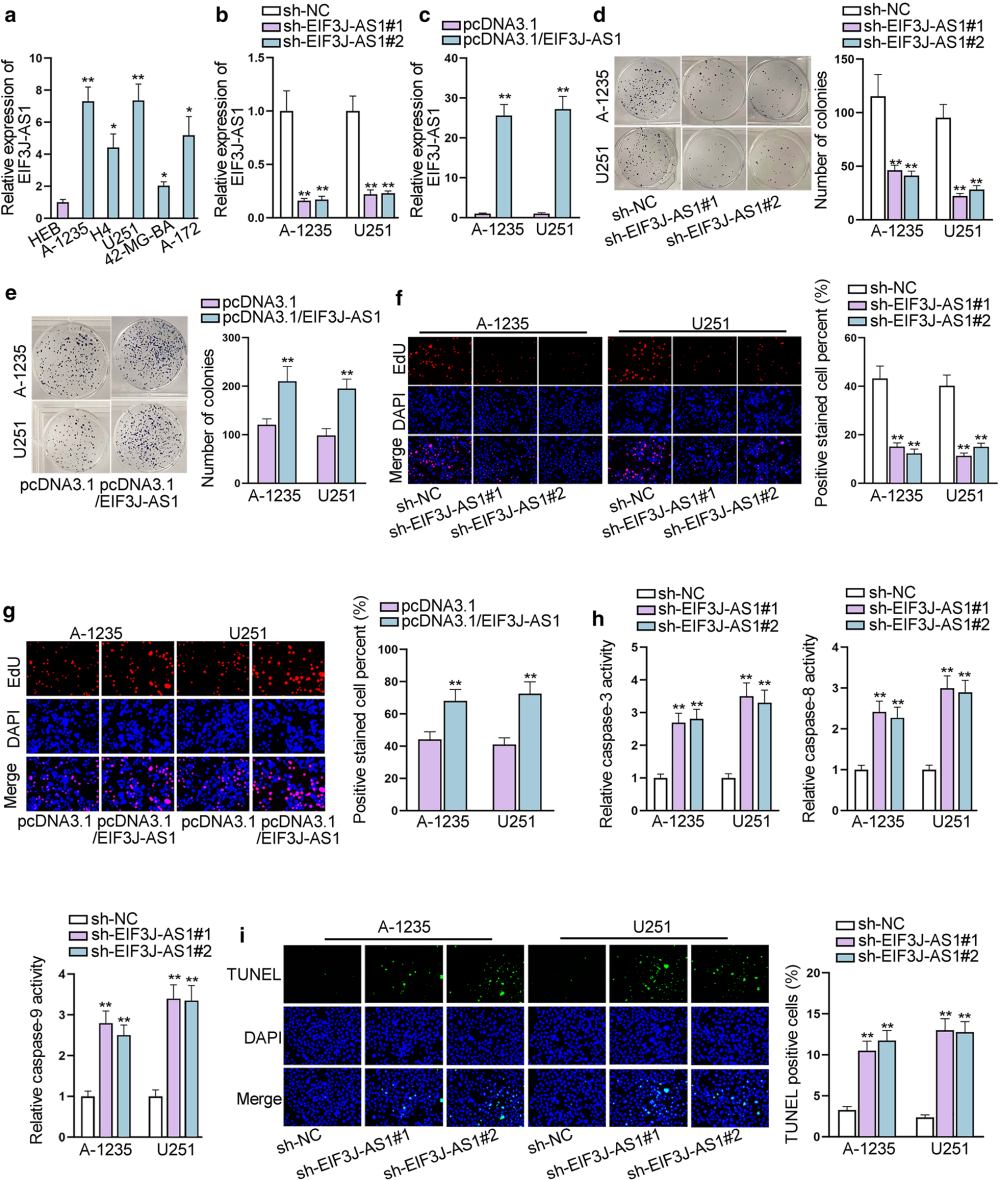
Upregulation of EIF3J-AS1 in glioma facilitates malignant progression of glioma. **a** The expression levels of EIF3J-AS1 in glioma versus normal human glial cell lines were measured by qRT-PCR analysis. **b**, **c** The inhibitory and overexpressing efficiency of EIF3J-AS1 was tested by qRT-PCR analysis. **d**, **e** Cells proliferation ability was assessed by colony formation after transfection of sh-EIF3J-AS1 or pcDNA3.1/EIF3J-AS1. **F**, **g** EdU assay was adopted to study the rate of positive EdU stained cell after transfection of sh-EIF3J-AS1 or pcDNA3.1/EIF3J-AS1. **h** TUNEL assay was performed to testify cell apoptosis. I. Caspase 3/8/9 activity assays were utilized to evaluate the activities of Caspase 3/8/9 respectively. *P < 0.05, **P < 0.01

To determine the possible role of EIF3J-AS1 in glioma, we evaluated its expression in 40 glioma samples with 10 normal brain tissues as control group. It was found that the level of EIF3J-AS1 exhibited relative high level in glioma patient samples (Additional file 1: Fig. S1A). Subsequently, we measured its expression in 5 glioma cell lines (A-1235, H4, U251, 42-MG-BA and A-172) and one normal glial cell line (HEB). EIF3J-AS1 expression levels in glioma cell lines were distinctively higher than the normal HEB cell line (Fig. 1a). We utilized A-1235 and U251 for further exploration of the function of EIF3J-AS1 in glioma, as EIF3J-AS1 was found most highly expressed in these two cell lines. Thus, specific shRNAs were designed and transfected into the above cell lines. Both shRNAs were proved to knock out the expression of EIF3J-AS1 effectively (Fig. 1b). We also constructed EIF3J-AS1 overexpression vector using pcDNA3.1, and found that EIF3J-AS1 was elevated by such transfection in selected glioma cells (Fig. 1c). Colony formation assay demonstrated that EIF3J-AS1 knockdown induced decreased colonies, while EIF3J-AS1 overexpression exerted the opposite outcome (Fig. 1d, e). EdU staining assay further confirmed that EIF3J-AS1 knockdown suppressed the proliferation ability of glioma cells, yet EIF3J-AS1 up-regulation promoted proliferation ability (Fig. 1f, g). Caspase-3/8/9 activity assay were adopted to testify the activity of Caspase 3/8/9 respectively. It turned that EIF3J-AS1 knockdown significantly enhanced the activity of Caspase 3/8/9 (Fig. 1h). Besides, TUNEL assay manifested that EIF3J-AS1 knockdown increased the ratio of TUNEL positive cells in A-1235 and U251 cells (Fig. 1i). In vivo animal study was conducted to demonstrate the role of EIF3J-AS1 in tumor growth. The tumor size, volume and weigh in sh-EIF3J-AS1 group were smaller than that in sh-NC group (Additional file 1: Fig. S1B–D). Based on these observations, we preliminarily supposed that EIF3J-AS1 may serve as an oncogene in glioma development.

### EIF3J-AS1 acts as a molecular sponge for miR-1343-3p in glioma

**Fig. 2:**
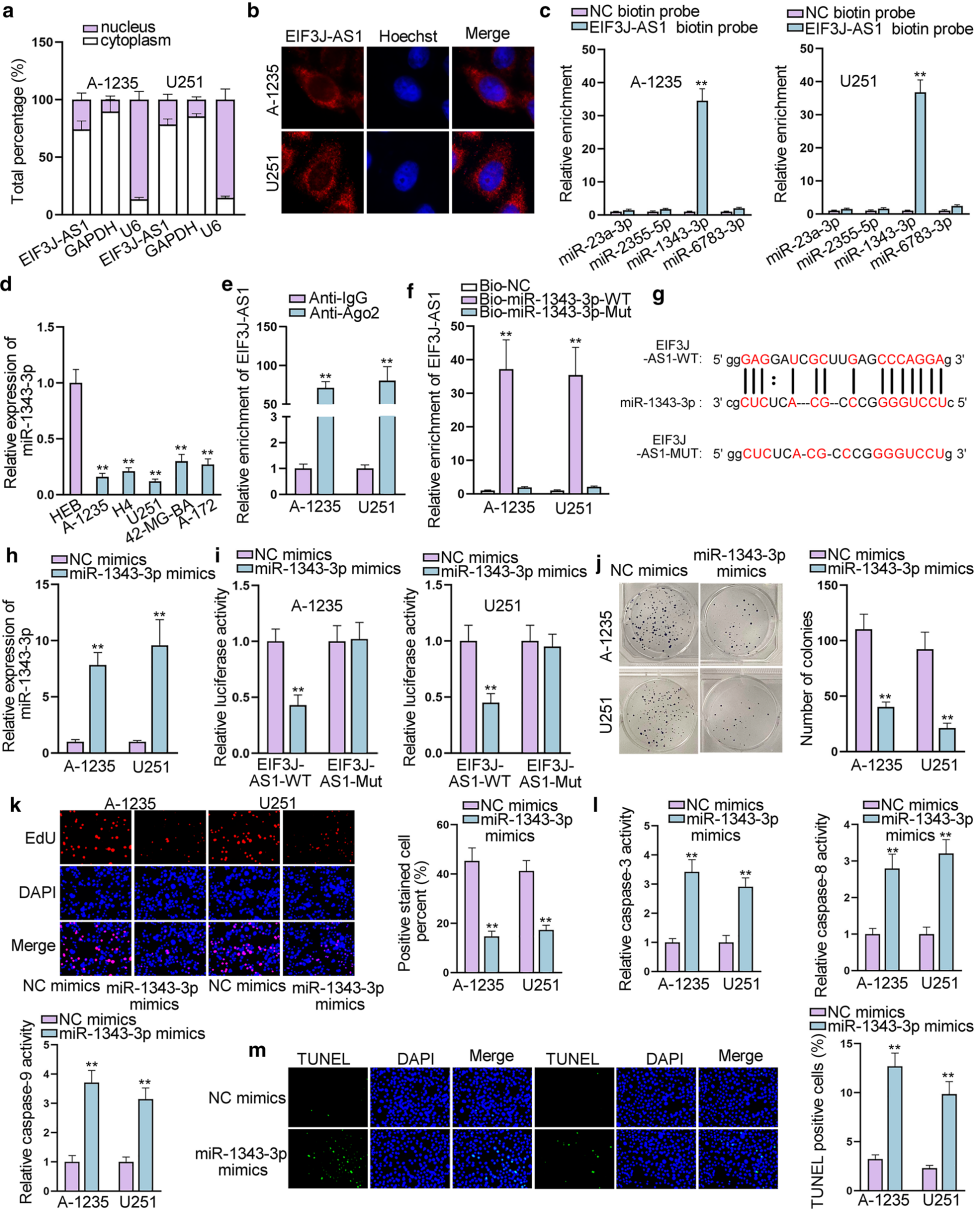
EIF3J-AS1 acts as a molecular sponge for miR-1343-3p in glioma. **a**, **b** Subcellular fractionation and FISH assays were combined to explore the location of EIF3J-AS1. **c** RNA pull down was performed to study the potential interaction between candidate miRNAs and EIF3J-AS1. **d** The expression of miR-1343-3p was detected in glioma cells and normal one. **e** RIP assay revealed that EIF3J-AS1 had the potential to bind to Ago2. **f** RNA pull down validated the interaction between EIF3J-AS1 and miR-1343-3p. **g** Putative and mutate miR-1343-3p binding sites with EIF3J-AS1 were displayed. **h** MiR-1343-3p expression was up-regulated by transfection of miR-1343-3p mimics. **i** Dual-luciferase reporters showed that miR-1343-3p mimics decreased the luciferase activity of vector containing EIF3J-AS1-WT. **j**, **k** Colony formation and EdU assays were performed to evaluate cell proliferation after up-regulating miR-1343-3p. **l**, **m** TUNEL and Caspase 3/8/9 activity assays were used to explore glioma cells apoptotic ability after up-regulating miR-1343-3p. **P < 0.01

To understand the potential regulatory mechanism of EIF3J-AS1, we first detected its subcellular situation in selected glioma cells. Subcellular fractionation and FISH assays showed that EIF3J-AS1 was localized primarily in the cytoplasm of A-1235 and U251 (Fig. 2a, b), indicating the post-transcriptional regulatory role of EIF3J-AS1. LncRNA can simultaneously bind with a same miRNA with mRNA, thus blocking the binding relation between miRNA and mRNA. In this process, lncRNA exert function as “miRNA sponge”. Using starBase database (http://starbase.sysu.edu.cn/), we screened out 4 potential miRNAs (miR-23a-3p, miR-2355-5p, miR-1343-3p and miR-6783-3p) sharing binding sites with EIF3J-AS1. RNA pull down assay was conducted to evaluate the combination between EIF3J-AS1 and these candidate miRNAs. Compared with other candidates, miR-1343-3p was found to be evidently enriched by biotin-labeled EIF3J-AS1 (Fig. 2c). Moreover, qRT-PCR showed that the expression level of miR-1343-3p was notably lower in 40 glioma tissues and 5 glioma cell lines than that of normal controls (Additional file 1: Fig. S1E and Fig. 2d). Importantly, the expression correlation between EIF3J-AS1 and miR-1343-3p was found to be negative (Additional file 1: Fig. S1F). Thus, we chose miR-1343-3p for further investigation. RIP assay revealed that EIF3J-AS1 was capable of binding to Ago2 protein (Fig. 2e). We then performed RNA pull down assay again utilizing biotin-labeled miR-1343-3p to pull down EIF3J-AS1. Its results showed a significant enrichment of EIF3J-AS1 by biotin-labeled miR-1343-3p (Fig. 2f). The potential binding sites of miR-1343-3p in the sequence of EIF3J-AS1 were predicted by online bioinformatics tool (Fig. 2g). Prior to dual-luciferase reporter assays, we overexpressed miR-1343-3p with the transfection of miR-1343-3p mimics (Fig. 2H). MiR-1343-3p mimics notably attenuated the relative luciferase activity of EIF3J-AS1-WT, which contained the wild type binding sequences for miR-1343-3p (Fig. 2i).

Given that the functional role of miR-1343-3p remains unknown in glioma, we performed functional assay. Colony formation and EdU assays manifested that miR-1343-3p overexpression restrained glioma cells proliferation (Fig. 2j, k). Apoptosis assays showed that miR-1343-3p overexpression enhanced apoptotic capacity in glioma cells (Fig. 2l, m). Together, these data showed that miR-1343-3p overexpression could curb the malignant behaviors of glioma cells.

### Inhibition of miR-1343-3p expression reverses the effects induced by EIF3J-AS1 knockdown

**Fig. 3:**
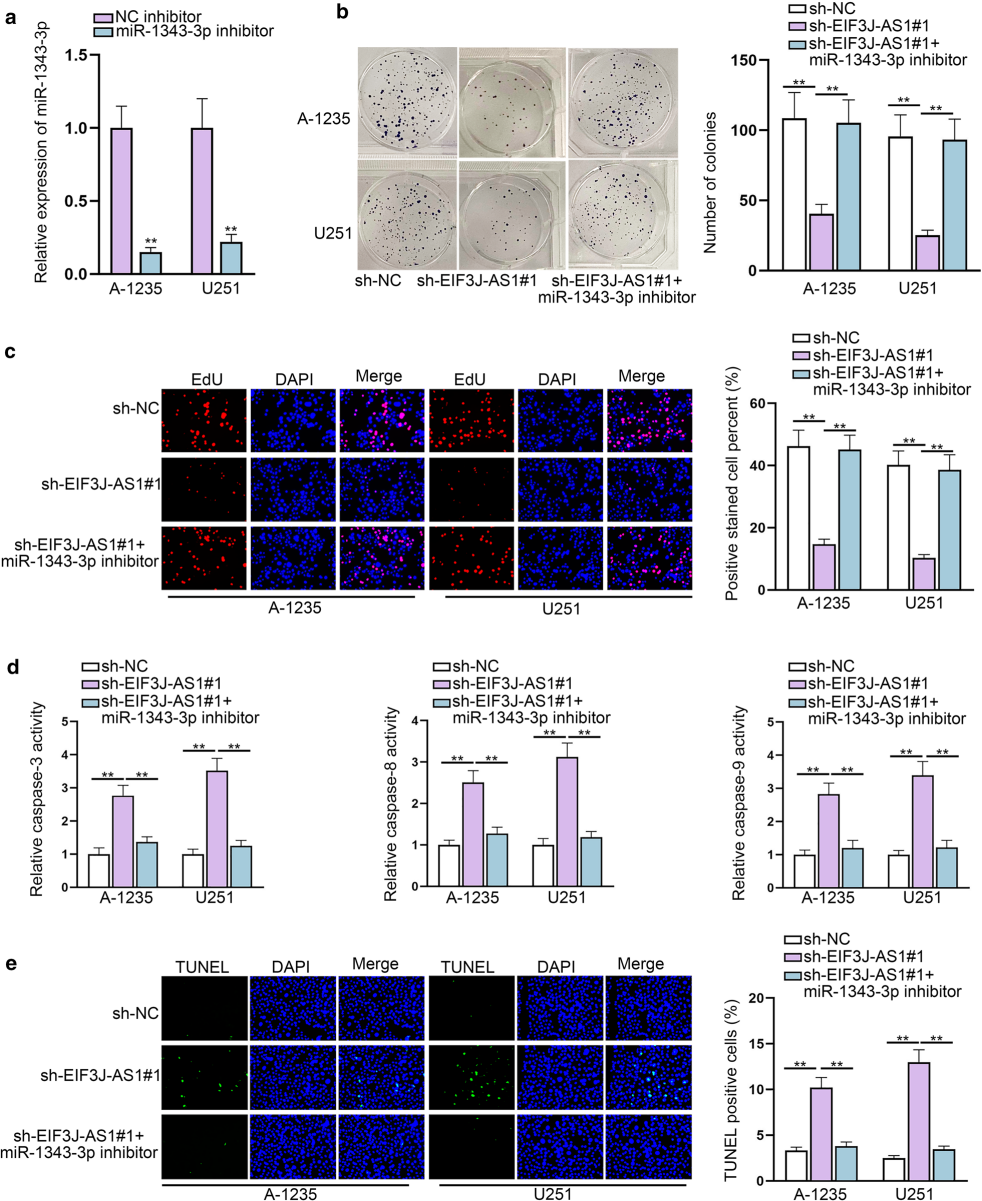
Inhibition of miR-1343-3p expression reverses the effects induced by EIF3J-AS1 knockdown. **a** The expression of miR-1343-3p was detected in after transfection of miR-1343-3p inhibitor in glioma cells. **b**, **c** The rescuing effects of miR-1343-3p on glioma proliferative ability were measured. **d**, **e** The rescuing impacts of miR-1343-3p on glioma apoptotic ability were explored. **P < 0.01

To testify whether EIF3J-AS1 induced effects on glioma via sponging miR-1343-3p, we assessed the effects of miR-1343-3p inhibitor on sh-EIF3J-AS1-mediated cellular functions. MiR-1343-3p expression was found depleted in responding to miR-1343-3p inhibitor in both A-1235 and U251 (Fig. 3a). We found that miR-1343-3p down-regulation dramatically countervailed the interfering proliferative (Fig. 3b, c) and strengthened apoptotic activities (Fig. 3d, e) by EIF3J-AS1 knockdown. These findings suggested that EIF3J-AS1 might sponge miR-1343-3p to elicited function in glioma cellular behaviors.

### ANXA11 is the downstream target of miR-1343-3p

**Fig. 4:**
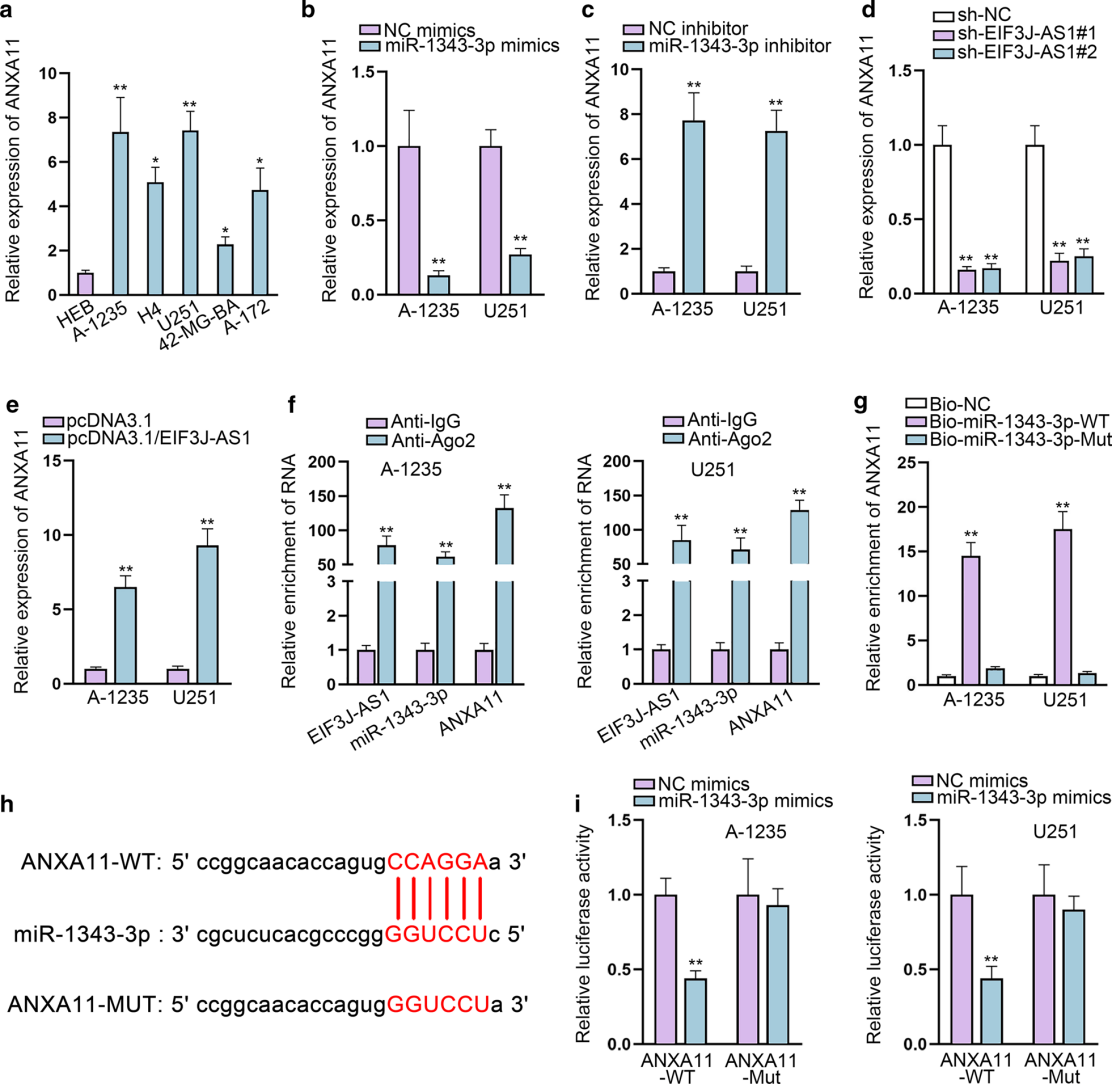
ANXA11 is the downstream target of miR-1343-3p. **a** The expression of ANXA11, the target of miR-1343-3p, was evaluated in glioma cell lines and normal glial HEB. **b**, **c** The expression of ANXA11 was detected by qRT-PCR after transfecting miR-1343-3p mimics or miR-1343-3p inhibitor. **d**, **e** The expression levels of ANXA11 were detected in response to sh-ANXA11 and pcDNA3.1/ANXA11. **f** RIP assay showed co-precipitated EIF3J-AS1, miR-1343-3p and ANXA11 by antibody targeting Ago2. **g** RNA pull down validated the interaction between ANXA11 and miR-1343-3p. **h** Putative and mutant binding sequences of miR-1343-3p in 3′UTR sequence of ANXA11. **i** Dual-luciferase reporters showed that miR-1343-3p mimics could impair the luciferase activity of ANXA11-WT. *P < 0.05, **P < 0.01

MiRNAs have been widely reported to interact with their targets and lead to translational repression or RNA degradation basically in an AGO2-dependent way [18]. To further identify the possible post-transcriptional role of EIF3J-AS1, we searched potential mRNAs that might bind to miR-1343-3p. StarBase bioinformatics website screened out ANXA11 as the most potential combinable mRNA for miR-1343-3p (CLIP-Data ≥ 1, Degradome-Data ≥ 3, Pan-Cancer ≥ 8, programNum ≥ 1). We then detected the expression of ANXA11 in glioma samples and cell lines. The results showed that ANXA11 exhibited relative high level in glioma samples and cell lines (Additional file 1: Fig. S1G and Fig. 4a). The expression association between ANXA11 and miR-1343-3p was negative (Additional file 1: Fig. S1H) but we analyzed the positive correlation between ANXA11 and EIF3J-AS1 (Additional file 1: Fig. S1I). Subsequent qRT-PCR showed that ANXA11 was down-regulated by miR-1343-3p mimics, while up-regulated by miR-1343-3p depletion (Fig. 4b, c). We performed qRT-PCR assay to detect whether EIF3J-AS1 elicited effects on ANXA11. It was found that EIF3J-AS1 positively regulated ANXA11 expression (Fig. 4d, e). RIP assay revealed that EIF3J-AS1, miR-1343-3p and ANXA11 were all enriched in Ago2-bound complexes (Fig. 4f). Furthermore, RNA pull down assay indicated that ANXA11 was enriched by biotin-labeled miR-1343-3p (Fig. 4g). StarBase database uncovered that ANXA11 shared binding sites for miR-1343-3p (Fig. 4h). Moreover, dual-luciferase reporter assays further proved the binding between ANXA11 and miR-1343-3p. It manifested that miR-1343-3p mimics effectively decreased the relative luciferase activity of ANXA11-WT, instead of ANXA11-Mut (Fig. 4i).

### ANXA11 silence induces the inhibition of glioma cell growth

**Fig. 5:**
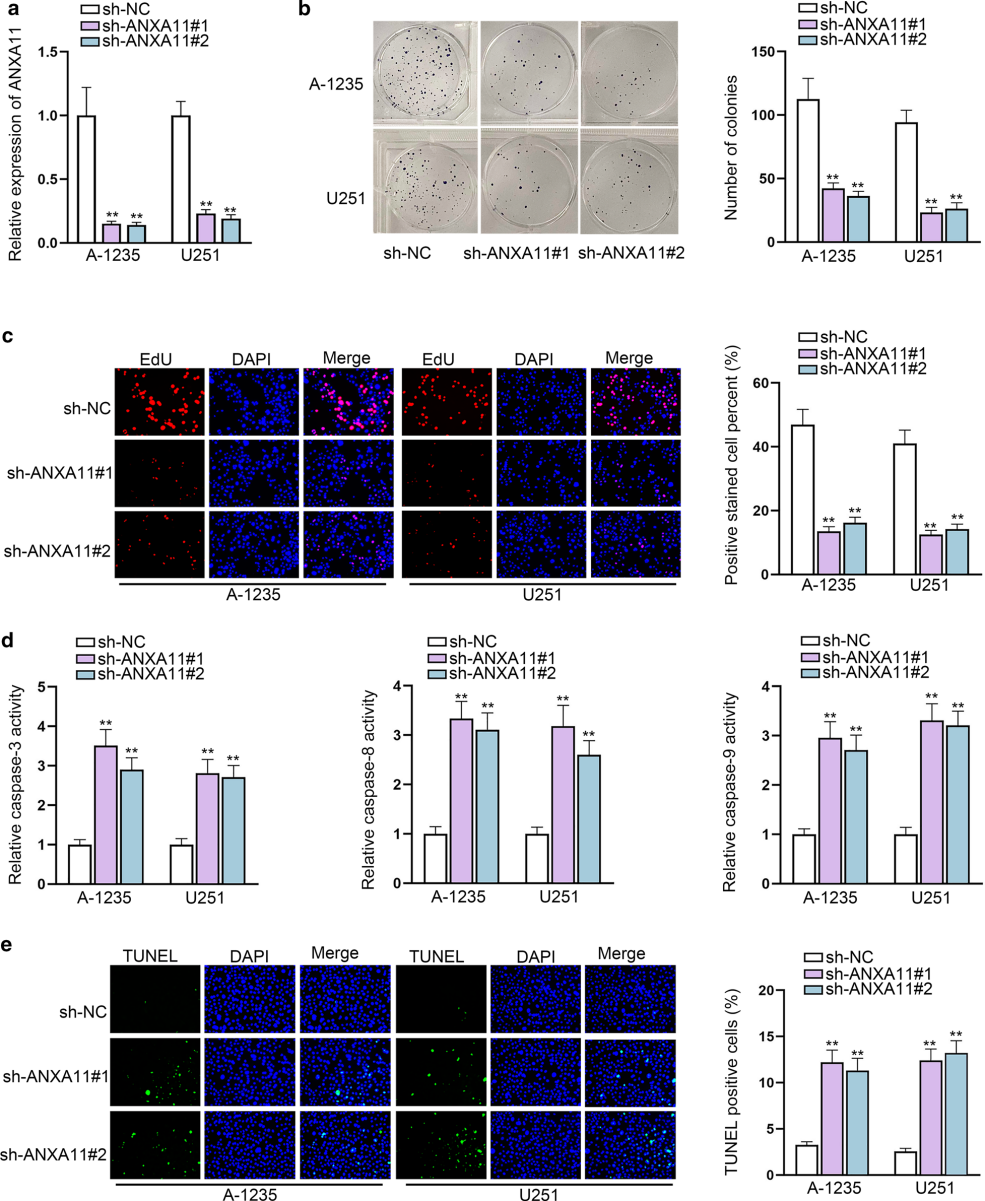
ANXA11 silence induces the inhibition of glioma cell growth. **a** The expression of ANXA11 was decreased after specific sh-ANXA11 transfection into glioma cells. **b**, **c** The role of silencing ANXA11 on glioma proliferative ability was studied by colony formation and EdU assays. **d**, **e** The role of silencing ANXA11 on apoptosis ability in glioma cells was investigated. **P < 0.01

ANXA11 is a kind of membrane-binding annexin proteins that has been found to be connected with the metastasis, invasion and drug resistance of cancers through PDGFR or MAPK/p53 pathway [19]. However, its role in glioma lacks intensive study. We knock out ANXA11 by transfecting sh-ANXA11 into glioma cells (Fig. 5a). ANXA11 silence significantly impaired the proliferative ability of glioma cells (Fig. 5b, c). Meantime, the apoptosis activity of glioma cells was facilitated by silencing ANXA11 (Fig. 5d, e). It can be deduced that ANXA11 knockdown exerted the similar outcomes of EIF3J-AS1 silencing in glioma development.

### ANXA11 is involved in EIF3J-AS1-mediated glioma cell functions

**Fig. 6:**
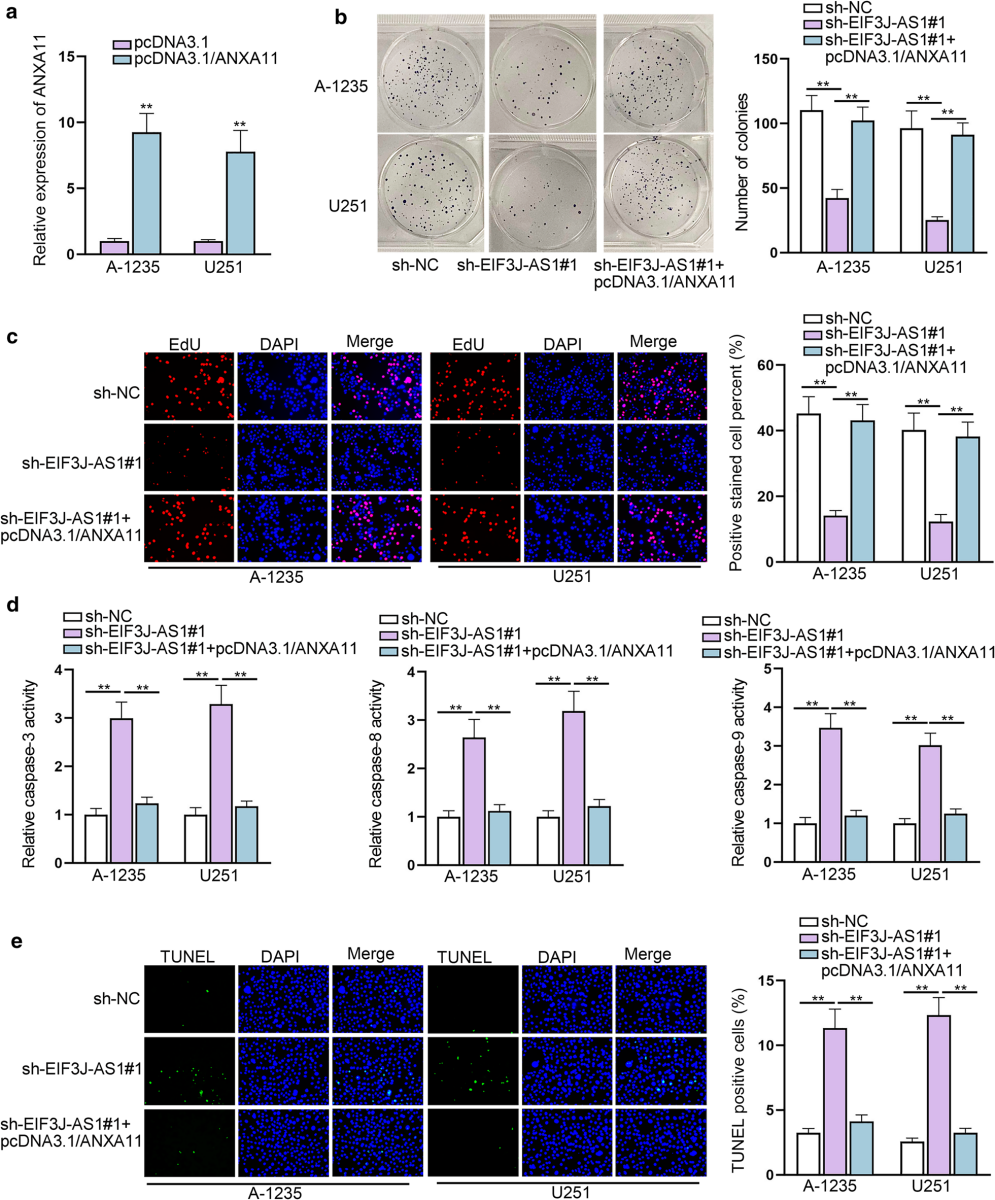
ANXA11 is involved in EIF3J-AS1-medited glioma cell functions. **a** The expression level of ANXA11 was evaluated by qRT-PCR after transfecting with pcDNA3.1/ANXA11. **b**, **c** Colony formation and EdU assays were combined to study the rescuing effect of pcDNA3.1/ANXA11 on sh-EIF3J-AS1 induced proliferation. **d**, **e** TUNEL and caspase 3/8/9 activity assays were utilized to study the rescuing effect of pcDNA3.1/ANXA11 on sh-EIF3J-AS1 induced apoptosis. **P < 0.01

To further testify the ceRNA network of EIF3J-AS1/miR-1343-3p/ANXA11 in glioma, we overexpressed ANXA11 to evaluate the biological behaviors induced by EIF3J-AS1 silencing. At first, the expression of ANXA11 was elevated by transfecting pcDNA3.1/ANXA11 (Fig. 6a). Both colony formation and EdU assays indicated that cell proliferative ability inhibited by silencing EIF3J-AS1 was recovered again by ANXA11 abundance (Fig. 6b, c). Moreover, the apoptosis ability was stimulated by EIF3J-AS1 silencing, yet hampered again by ANXA11 abundance (Fig. 6d, e). These tendencies on proliferation and apoptosis were identical with those miR-1343-3p inhibitor induced outcomes on EIF3J-AS1-depleted glioma cells. Hence, we concluded that EIF3J-AS1 promoted proliferation, while inhibiting apoptosis of glioma cells via positively regulating ANXA11 through sponging miR-1343-3p.

## Discussion

As a lethal tumor that originated form central nervous system, glioma posed a significant burden to the health of patients [20]. Aberrant expression of RNA molecules was proven to be relevant in the tumorigenesis of glioma [21]. LncRNAs has been reported to participate in glioma progression [22]. LncRNA could act as specific miRNA sponge and induce activation of miRNA-mediated cancer-associated signaling pathways, such as Wnt/β-catenin and PI3K/AKT/mTOR [23]. The novel lncRNA EIF3J-AS1 was elucidated to aggravate cells proliferation and inhibit apoptosis in colorectal cancer via up-regulating YAP1 through sequestering miR-316 [24]. In our study, EIF3J-AS1 was up-regulated in glioma tissues and cells. Consistently, we determined the oncogenic role of EIF3J-AS1 in glioma by promoting glioma cell growth and blocking cell apoptosis.

It is known that cytoplasmic lncRNAs can interact with miRNA to block miRNA-induced inhibition on mRNA. For instance, lncRNA SNHG3 could positively regulated RAB22A via sequester miRNA-151a-3p [25]. In our current study, we unveiled that miR-1343-3p is the downstream miRNA that could interact with EIF3J-AS1. MiRNAs referred to a subtype of non-coding RNA transcripts about 22 nucleotides in length and mainly inversely regulate the expression of protein-coding genes [18]. The investigation on the biological performance of miRNA has been relatively well-documented. For instance, miR-122-5p impeded cell migration and invasion by negatively modulating DUSP4 in gastric cancer [26]. Down-regulated miR-1343-3p was discovered to be associated with multiple cancer types, suggesting miR-1343-3p as potential candidate for disease biomarkers [27]. MiR-1343-3p expression pattern appeared to exhibit distinctive clinicopathological implications in ALK-rearranged lung adenocarcinoma [28]. Functionally, we identified that upregulation of miR-1343-3p significantly suppressed cell growth in glioma, suggesting that miR-1343-3p might exert anti-oncogenic role in glioma. MiR-1343-3p inhibitor offset the cellular behaviors mediated by EIF3J-AS1 silence, Hereto, our study indicated that EIF3J-AS1 positively regulated glioma cell growth through interacting with miR-1343-3p.

MiRNA primarily induce function via downstream target mRNAs in biological process. Here, ANXA11 was proven to be the target mRNA of miR-1343-3p in glioma. According to a previous report, ANXA11 was abnormally expressed in HCC tissues and cell lines, and was positively regulated by lncRNA AGAP2-AS1 via sharing a common miRNA miR-16-5p [29]. In this study, we firstly detected the correlation between miR-1343-3p and ANXA11 in glioma and analyzed their association with lncRNA EIF3J-AS1. Importantly, ANXA11 silence led to the suppression of glioma cell growth. All our findings indicated that EIF3J-AS1, miR-1343-3p and ANXA11 constituted a novel ceRNA pathway, which was responsible for the glioma growth. Our research findings revealed the role of the new-found lncRNA EIF3J-AS1 in glioma and explored a novel downstream pathway of EIF3J-AS1. We provided in vitro and in vivo data and analyzed the clinical significance. Lack of deep investigation of the upstream molecular mechanism of EIF3J-AS1 is a limitation of our current study. We will explore and analyze the deep upstream molecular mechanism of EIF3J-AS1/miR-1343-3p/ANXA11 axis in our future work.

## Conclusion

In short, our study put forward a novel ceRNA pathway EIF3J-AS1/miR-1343-3p/ANXA11 in glioma and detected the functions of them in glioma growth. All findings in this study may provide potential novel therapeutic target for glioma.

## Supplementary information

**Additional file 1: Figure S1.** A. EIF3J-AS1 expression in 10 normal tissues and 40 glioma tissues. B. Tumors removed from mice in sh-NC group or sh-EIF3J-AS1 group were shown. C. Tumor volume in each group was calculated and recorded. D. Tumors removed from different groups were weighted. E. The level of miR-1343-3p was evaluated in glioma samples compared to normal samples. F. The expression correlation between EIF3J-AS1 and miR-1343-3p. G. ANXA11 expression in 10 normal tissues and 40 glioma tissues. H. The correlation between miR-1343-3p and ANXA11 in glioma tissues. I. The expression correlation between EIF3J-AS1 and ANXA11 and EIF3J-AS1. **P < 0.01.

## Data Availability

Not applicable.

## Abbreviations

lncRNAs: long non-coding RNAs
EIF3J-AS1: EIF3J antisense RNA 1
ANXA11: Annexin A11
RISC: RNA induced silencing complex
ceRNA: competing endogenous RNA
MREs: MiRNA-response elements
mRNAs: messenger RNAs
qRT-PCR: quantitative reverse transcription polymerase chain reaction
FISH: fluorescence in situ hybridization
RIP: RNA Immunoprecipitation
ceRNA: competing endogenous RNA
