# Supplementary figures and images for: EIF3J-AS1 promotes glioma cell growth via up-regulating ANXA11 through sponging miR-1343-3p
Source: Cancer Cell Int. 2020 Sep 3;20:428. doi: 10.1186/s12935-020-01487-2 (PMC7469350; doi:10.1186/s12935-020-01487-2)

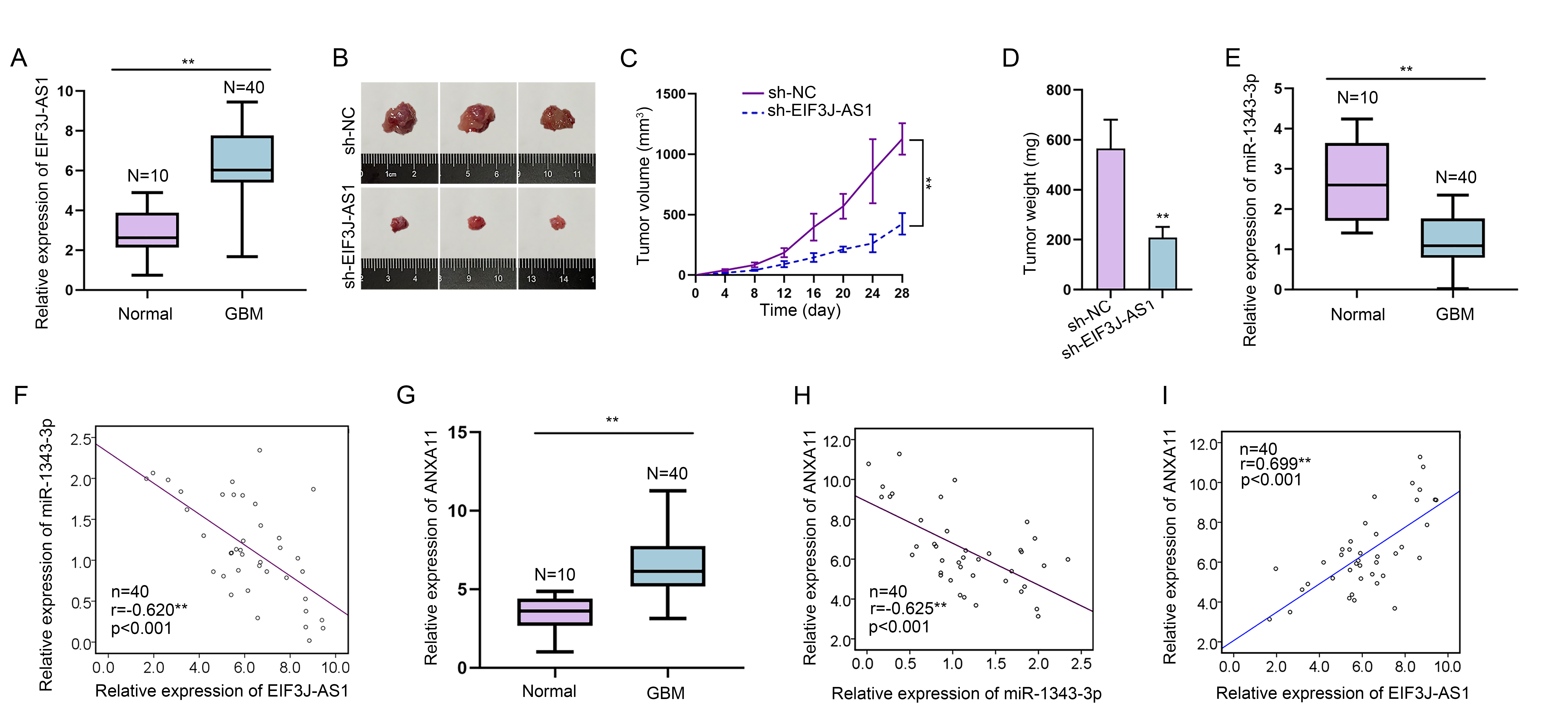

Supplement: Supplementary file 1 — Additional file 1: Figure S1. A. EIF3J-AS1 expression in 10 normal tissues and 40 glioma tissues. B. Tumors removed from mice in sh-NC group or sh-EIF3J-AS1 group were shown. C. Tumor volume in each group was calculated and recorded. D. Tumors removed from different groups were weighted. E. The level of miR-1343-3p was evaluated in glioma samples compared to normal samples. F. The expression correlation between EIF3J-AS1 and miR-1343-3p. G. ANXA11 expression in 10 normal tissues and 40 glioma tissues. H. The correlation between miR-1343-3p and ANXA11 in glioma tissues. I. The expression correlation between EIF3J-AS1 and ANXA11 and EIF3J-AS1. **P < 0.01. [file 12935_2020_1487_MOESM1_ESM.tif]
